# Rapid decrease in tumor perfusion following VEGF blockade predicts long-term tumor growth inhibition in preclinical tumor models

**DOI:** 10.1007/s10456-012-9328-3

**Published:** 2012-12-13

**Authors:** Alexandra Eichten, Alexander P. Adler, Blerta Cooper, Jennifer Griffith, Yi Wei, George D. Yancopoulos, Hsin Chieh Lin, Gavin Thurston

**Affiliations:** Regeneron Pharmaceuticals Inc, 777 Old Saw Mill River Road, Tarrytown, NY 10591 USA

**Keywords:** VEGF blockade, Tumor perfusion, Tumor growth response, Preclinical model, Response biomarker

## Abstract

Vascular endothelial growth factor (VEGF) is a key upstream mediator of tumor angiogenesis, and blockade of VEGF can inhibit tumor angiogenesis and decrease tumor growth. However, not all tumors respond well to anti-VEGF therapy. Despite much effort, identification of early response biomarkers that correlate with long-term efficacy of anti-VEGF therapy has been difficult. These difficulties arise in part because the functional effects of VEGF inhibition on tumor vessels are still unclear. We therefore assessed rapid molecular, morphologic and functional vascular responses following treatment with aflibercept (also known as VEGF Trap or ziv-aflibercept in the United States) in preclinical tumor models with a range of responses to anti-VEGF therapy, including Colo205 human colorectal carcinoma (highly sensitive), C6 rat glioblastoma (moderately sensitive), and HT1080 human fibrosarcoma (resistant), and correlated these changes to long-term tumor growth inhibition. We found that an overall decrease in tumor vessel perfusion, assessed by dynamic contrast-enhanced ultrasound (DCE-US), and increases in tumor hypoxia correlated well with long-term tumor growth inhibition, whereas changes in vascular gene expression and microvessel density did not. Our findings support previous clinical studies showing that decreased tumor perfusion after anti-VEGF therapy (measured by DCE-US) correlated with response. Thus, measuring tumor perfusion changes shortly after treatment with VEGF inhibitors, or possibly other anti-angiogenic therapies, may be useful to predict treatment efficacy.

## Introduction

Vascular endothelial growth factor (VEGF) plays a key role in physiological and pathological angiogenesis, including tumor angiogenesis [1]. Therefore, a number of agents that inhibit VEGF signaling have been developed and tested in clinical trials [2, 3]. Bevacizumab, a VEGF specific antibody that prevents receptor binding and activation, slowed tumor progression and provided survival benefits in several human tumor types when used in combination with chemotherapy. In addition, several small molecule inhibitors of VEGF receptor tyrosine kinase activity provided benefit in various cancers [4–6]. In preclinical models, VEGF inhibition results in reduced tumor growth, decreased microvessel density (MVD) and normalization of tumor vessel morphology in a wide range of tumor types [7, 8]. Similar MVD reductions were also reported in clinical studies of colorectal tumors sampled shortly after bevacizumab treatment [9].

Despite clear evidence for tumor vessel loss following VEGF inhibition, the functional consequences on tumor blood flow and oxygenation are not entirely clear. Naïvely, one might expect that vessel loss would result in decreased tumor perfusion. However, more detailed considerations suggested the opposite, namely, that tumor vessel pruning and “normalization” may lead to decreased intra-tumoral pressure, increased tumor perfusion, and consequently decreased tumor hypoxia [10]. Indeed, some preclinical studies indicate increased tumor perfusion after VEGF blockade [11, 12]. In contrast, other studies have reported increased tumor hypoxia and decreased perfusion in preclinical models and non-small cell lung cancer (NSCLC) patients [13–15]. Thus, the functional consequences of anti-VEGF therapy are not clear, even in preclinical tumor models.

To add to the complexity, not all tumors within a given tumor type respond equally well to anti-VEGF therapy. For example, in glioblastoma patients treated with a small molecule kinase inhibitor (cediranib), approximately 60 % of tumors displayed changes in dynamic contrast-enhanced magnetic resonance imaging (DCE-MRI) signals indicative of a response to anti-VEGF therapy, whereas the remaining 40 % did not [16]. Despite much effort, predicting which tumors will respond to anti-VEGF therapy, or how long-term tumor growth response is related to vascular changes, has been difficult. For instance, it is unknown whether tumors with the largest MVD reduction show the greatest tumor growth inhibition (TGI). Further, tumor vessel features rendering them sensitive, or resistant, to VEGF inhibition are not well understood. Ultimately, predictive biomarkers based on mechanistic differences in tumor cells and tumor blood vessels are needed.

To begin to address these issues, we characterized initial responses of tumor vessels to VEGF blockade in preclinical tumors with a range of responses to anti-VEGF therapy (sensitive, moderately responsive, and resistant). For these studies, we used aflibercept (also known as VEGF Trap or ziv-aflibercept in the United States), a recombinant fusion protein that potently binds all isoforms of human and murine VEGF-A, VEGF-B and Placental Growth Factor (PlGF). Tumor bearing mice were treated with aflibercept, and tumors were analyzed for rapid (within 3 days) changes in molecular (gene expression), morphologic (MVD) and functional (vascular perfusion, tumor hypoxia) tumor vessel properties. These changes were then compared to aflibercept-mediated longer-term tumor growth effects. Using this approach, we observed that functional changes correlated well with the overall level of TGI, whereas molecular or morphological changes showed a poor correlation. These findings suggest that changes in functional parameters, such as tumor perfusion and hypoxia, may be good predictors of long term growth inhibition.

## Materials and methods

### In vivo tumor studies

Animal studies were performed in accordance with Regeneron’s Institutional Animal Care and Use Committee guidelines. Tumor cells were obtained from the American Type Culture Collection (ATCC), except for the PC3 M line, which was obtained from the NCI, DCT Tumor Repository, NCI-FCRF, Frederick, MD. 1 × 10^6^ Colo205 human colon carcinoma, 1 × 10^6^ C6 rat glioblastoma, 2 × 10^6^ HT1080 human fibosarcoma, 1 × 10^6^ A431 human squamous cell carcinoma, 1 × 10^6^ 786-0 human renal cell carcinoma, 5 × 10^5^ MMT murine mammary carcinoma, 1 × 10^6^ PC-3 M metastasis-derived variant of human prostate adenocarcinoma PC-3 and 1 × 10^6^ LLC murine Lewis lung carcinoma cells were grown s.c. in male CB.17/SCID mice (Taconic). When tumors reached approximately 100 mm^3^, mice were treated by s.c. injection with hFc (control protein, 25 mg/kg) or a maximally effective anti-tumor dose of aflibercept [17] (VEGF Trap, ziv-aflibercept, 25 mg/kg) (# mice per treatment group: n = 5–7 tumor growth; n = 4–5 IHC; n = 3–4 TaqMan; n = 8–24 micro-ultrasound; n = 5–10 FITC-lectin flow cytometry). For long-term studies treatments occurred 2 × per week. Mice were monitored for tumor growth and overall health. HypoxyProbe-1 (Chemicon; 60 mg/kg) was injected i.p. 1 h prior to sacrifice. Tumors were harvested: ~½ tumor in 4 % paraformaldehyde, a cross-section in OCT, ~½ tumor in RNAlater. % Tumor Growth Inhibition (TGI) was calculated as follows: [1 − ((T_final_ − T_initial_)/(C_final_ − C_initial_))]*100, where T = aflibercept-treated tumor volumes and C = control-treated tumor volumes at treatment start and after 10-14 day treatment (10 days: LLC, MMT; 14 days: HT1080, Colo205, C6, A431, 786-0, PC-3 M). Tumor growth curves are presented as mean ± standard error of the mean (SEM).

### Immunohistochemistry and image analysis

IHC on gelatin embedded tissue sections: Tissues were fixed in 4 % paraformaldehyde for 72 h and embedded in a 4 % gelatin/PBS solution. Gelatin blocks were fixed in 4 % paraformaldehyde overnight at 4 °C, then transferred into a 30 % sucrose/PBS solution at 4 °C until the blocks sunk (~72 h). Tissue was cut into 80 μm sections, which were stored in cryoprotectant (1 % Polyvinylpyrrolidone, 30 % glycerol, and 30 % sucrose in NaPBS) at −20 °C until further use. For IHC detection of CD31 and HypoxyProbe (pimonidazole), sections were treated as follows: 30 min in 0.3 % H_2_O_2_ at 4 °C, 2 h in blocking solution (CD31: 0.3 % Triton X100/4 % normal rabbit serum/1 % BSA/PBS; HypoxyProbe: 0.3 % Triton X100/4 % normal horse serum/1 % BSA/PBS) at RT followed by an overnight incubation at 4 °C with rat anti-murine CD31 Ab (1:150; BD; MEC13.3) or a mouse anti-HypoxyProbe-1 antibody (1:1,000; Chemicon) diluted in the respective blocking solution containing 1 % serum. After five 3 min washes in PBS, CD31 was detected with a biotinylated mouse-adsorbed rabbit anti-rat antibody (1:150; Vector Laboratories;) and HypoxyProbe was detect with a biotinylated horse anti-mouse antibody (1:500; Vector Laboratories;) in a 2 h incubation at RT. Sections were subjected to an ABC reaction according to the manufacturers recommendations (Vector Laboratories; ABC VectaStain Elite) for 1 h at RT diluted in 1 % BSA in 50 mM PBS. After five 3 min washes in PBS, antigens were revealed with 3,3’-diaminobenzidine (DAB, Sigma).

OCT embedded tumors were cut into 30 μm frozen sections. Tissue was air dried, 10 min fixed in acetone (−20 °C), avidin–biotin blocked (Vector), blocked in 2.5 % normal goat serum/1 % BSA/PBS for 30–45 min (RT), incubated for 16 h at 4 °C with rat anti-murine CD31 Ab (1:50; BD) diluted in 0.5 × block followed by a 45 min (RT) incubation with a biotinylated anti-rat antibody (1:150; Vector). Antigens were revealed with 3,3’-diaminobenzidine (DAB, Sigma).

For analysis photomicrographs were acquired at 2.5 × magnification. Vessel density and hypoxia area were determined using NIH image software as previously described [18].

### RNA preparation and TaqMan analysis

Total RNA was purified using RNeasy (Qiagen). RNA quality and concentration were evaluated using a spectophotometer (NanoDrop ND-1000). cDNA was synthesized using 1 μg of total RNA and High Capacity RNA to cDNA Mastermix Kit (ABI). Expression of various genes was normalized to cyclophilin expression. TaqMan primer and probe sequences are as follows:

**Table:** 

Gene	Forward primer	Reverse primer	Probe sequence
*mKcne3*	AGACCTGGTACATGAGCCTCCAT	CAAGTGACTGTGAAGGGTTGTGTT	TGGGCAGTCTCATCCT
*mNid2*	CCGCTGTGGCCCTAATTCT	TGCGGCATTCACACCTGTA	TGTGTGTCAACTTGGTGGG
*mCdh5*	AATCGGGAGCATGCCAAGT	TGGGCACCCCGTTGTC	CCCGTGCTCATCTC
*mTie1*	AGCCTGAGCCCTTGAGTTACC	AAAGTTGCCCTCCCCTATGAG	TGGGAGGACATCACC
*mRobo4*	GCTAGGCGCTTTCCATCCA	GCGGCTGCAGAGACTATCTGA	TTGGCTGGAACCTC
*mEsm1*	TCTGGACTTTCCCTTCTTCCAG	CTGTGTGGGAGGCAGAGGTC	TGCAGCAGCCAAATCTCCCAGCA
*mVegfA*	GTATGGCTGGCTGGGTCACT	GTTTGATCCGCATGATCTGTAGAG	ACCACTGTGATCTGC
*mCyclophilin*	CGTGGGCTCCGTCGTC	CCCTTCTTCTTATCGTTGGCC	TTGCTGCCCGGACCCTCCG

### Flow cytometry

Tumor bearing mice (C6 or HT1080 tumors, 100 mm^3^), or non-tumor control mice, were treated s.c. with hFc (control protein, 25 mg/kg) or aflibercept (VEGF Trap, 25 mg/kg) 24 h prior to tissue harvest. To label endothelial cells of functional vessels, mice were i.v. injected with FITC-conjugated *Lycopersicon esculentum* (tomato) lectin (2.0 mg/ml; Vector) 3 min prior to tissue harvest. Single cell suspensions were prepared from normal skin (n = 4, n = 2 no FITC-lectin), C6 tumors (n = 7 control or aflibercept, n = 4 no FITC-lectin) or HT1080 tumors (n = 7 control or aflibercept, n = 3 no FITC-lectin) as described previously [19] and endothelial cells were detected using a PE-conjugated anti-CD31 Ab (1:200; BD). DAPI (1 μg/ml; Invitrogen) was used to exclude dead cells. Data acquisition: Beckman-Coulter MoFlo Legacy; data analysis: FlowJo software (Tree Star). Data shown represent mean ± standard error of the mean (SEM).

### Dynamic contrast-enhanced micro-ultrasound (DCE-micro US)

Animals were anaesthetized (isofluorane (3.0 %)/medical air mixture), secured to heated platform and dehaired. Ultrasound gel (Aquasonic, Parker Laboratories) provided coupling interface between ultrasound probe and animal. Image acquisition: Vevo2100 micro-ultrasound imaging system (VisualSonics); contrast agent: MicroMarker™ (microbubbles, VisualSonics). Contrast agent was prepared with a final concentration of 2 × 10^9^ microbubbles/ml saline and a 50 μl bolus was delivered via tail vein catheter during image acquisition. Quantification of relative blood volume, which represents tumor perfusion, was determined by analysis of a 2D area representing the largest tumor cross-section (Vevo2100 analysis software).

### Statistical analyses

Statistical analyses were performed using Prism software. Specific test include 2-way ANOVA with Bonferroni post hoc test (tumor growth curves), 1-way ANOVA with Bonferroni post hoc test (vessel density, gene expression changes, hypoxia analysis) and Mann–Whitney test (micro-ultrasound analysis). *p* values <0.05 were considered statistically significant.

## Results

### Vessel morphology changes in tumors with a range of responses to aflibercept

**Fig. 1:**
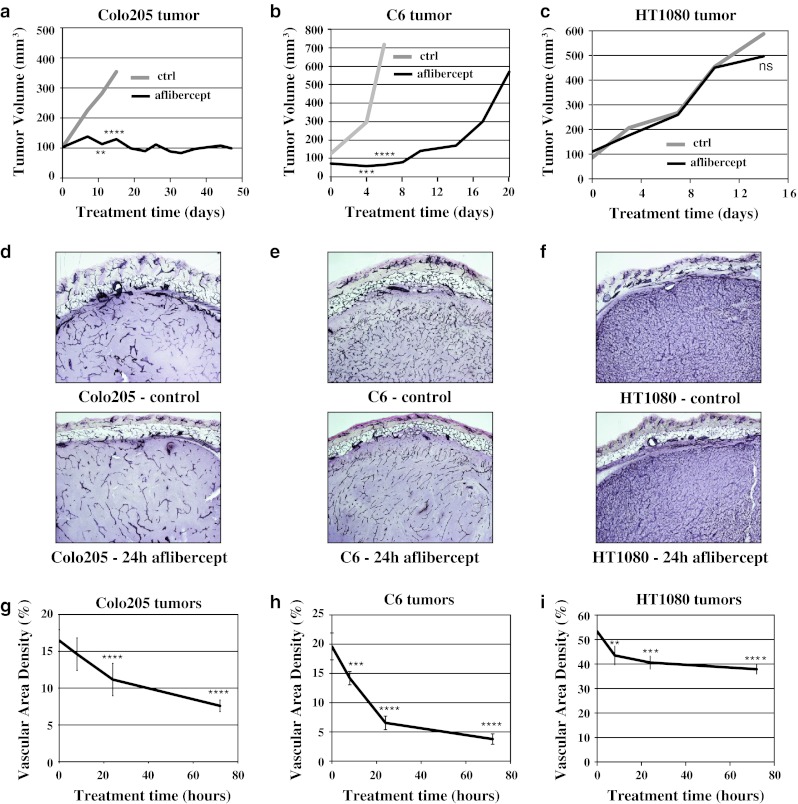
Tumor growth and vascular response to aflibercept in Colo205, C6 and HT1080 tumors. **a**–**c** Colo205, C6 and HT1080 xenografts (n = 5–7 each treatment group/tumor type) show different levels of TGI in response to aflibercept treatment (*black*) compared to control-treated tumors (*grey*): sensitive Colo205, moderately responsive C6 and resistant HT1080. **d**–**f** Representative images of MVD assessed by CD31 IHC in control and 24 h aflibercept-treated Colo205, C6 and HT1080 tumors (80 μm gelatin sections). **g**–**i** Quantitative analysis of vessel area density (%) in control and 8, 24 and 72 h aflibercept-treated Colo205, C6 and HT1080 tumors (n = 4–5 each time point/tumor type). All experiments were repeated at least twice; shown is an example experiment (n = 5–7 for each treatment group (tumor growth data) or n = 4–5 for each time point (MVD data)). Results shown represent means for tumor growth data and mean ± standard deviation (SD) for MVD analysis. *P* < 0.05*, <0.01**, <0.001***, <0.0001**** by 2 way-ANOVA with Bonferroni post hoc test (tumor growth data compared to control treated tumor growth) and by 1 way-ANOVA with Bonferroni post hoc test (MVD, each time point compared to control (0 time point))

Based on studies with a wide variety of murine tumor models, three tumors that display a range of responses to aflibercept were chosen for more detailed study. Colo205 tumors were potently growth inhibited (Fig. 1a), C6 tumors showed an intermediate growth inhibition in response to aflibercept treatment, with an initial growth delay followed by restrained tumor growth (Fig. 1b). In contrast, HT1080 tumors showed no growth inhibition upon aflibercept treatment (Fig. 1c). These differences in tumor response were observed at a saturating dose of aflibercept (25 mg/kg twice per week), thus the differences reflect inherent responses to aflibercept and not merely different dose responses.

We investigated the rapid effects of VEGF blockade on the vasculature of these 3 tumor types. As revealed by immunohistochemistry (IHC) for the vessel specific marker CD31 in thick sections, untreated Colo205 and C6 tumors have a significantly lower MVD (17 and 20 %, respectively) than HT1080 tumors (55 %) (Fig. 1d–f upper images; g–i time point 0). Following aflibercept treatment, MVD rapidly decreased in all tumor types, albeit to varying degrees (Fig. 1d–f lower images; g–i). Quantitative analysis of relative MVD after aflibercept administration in comparison to control-treated tumors revealed that Colo205 tumors lost 11, 32 and 54 % of their vasculature at 8, 24 and 72 h after treatment, respectively. C6 tumors lost even more vessels (28, 67 and 81 % at 8, 24 and 72 h after treatment, respectively). Aflibercept resistant HT1080 tumors progressively lost vessels after aflibercept treatment, albeit to a much lesser degree (up to 29 % by 72 h), suggesting that the HT1080 tumor vasculature is only partially dependent on VEGF. These results show that blockade of VEGF can cause rapid loss of tumor vascularity, and further, that the vasculature in different xenograft tumors varies in its dependence on ongoing VEGF signaling.

### Identification of two phases of gene expression changes in tumor vessels following aflibercept treatment

**Fig. 2:**
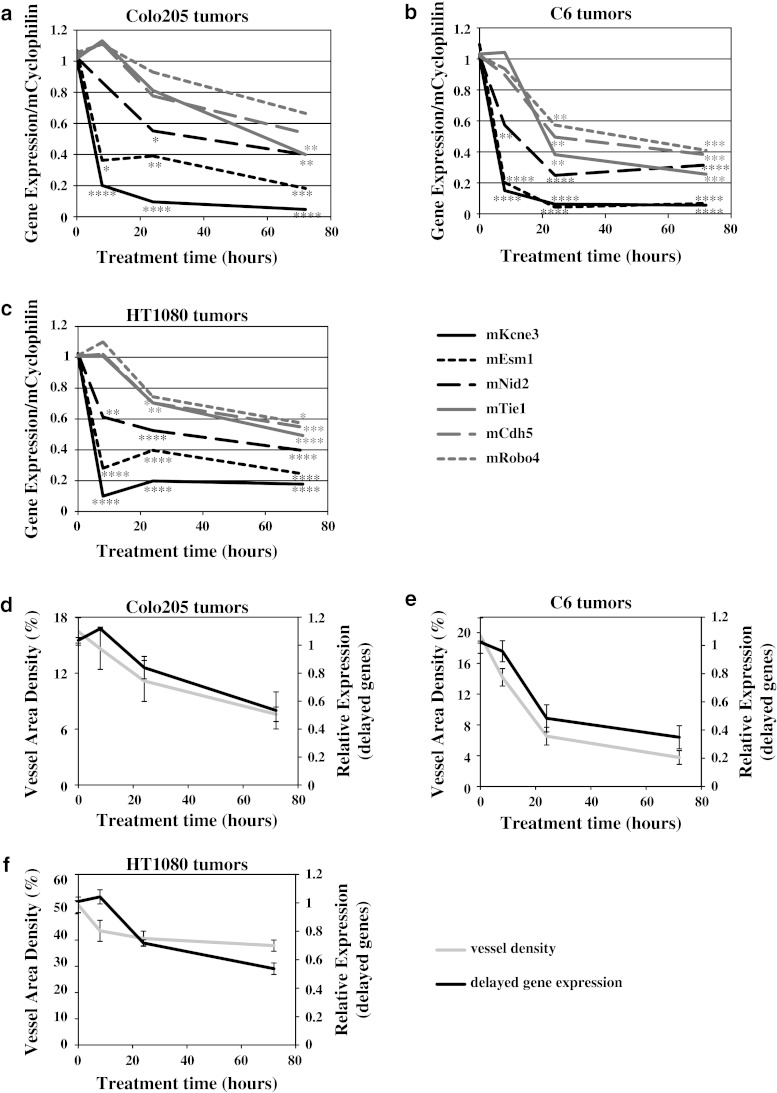
Gene expression analysis revealed two phases of gene expression changes in tumor vessels following aflibercept treatment. **a**–**c** Gene changes (TaqMan) upon VEGF blockade occur in two distinct patterns: ‘acute’ gene changes occur in Kcne3, Esm1 and Nid2 (*black*), while ‘delayed’ gene changes occur in Tie1, Cdh5 and Robo4 (*grey*) in Colo205, C6 and HT1080 tumors after 8, 24 and 72 h aflibercept treatment (n = 3–4 each time point/tumor type) **d**–**f** Averages of MVD changes (*grey*) and averages of ‘delayed’ gene expression changes (*black*) show a corresponding pattern in Colo205, C6 and HT1080 tumors after 8, 24 and 72 h aflibercept treatment. All experiments were repeated at least twice; shown is an example experiment for TaqMan data (n = 3–4 for each time point). Results shown represent means or mean ± standard deviation (SD). *P* < 0.05*, <0.01**, <0.001***, <0.0001**** by 1 way-ANOVA with Bonferroni post hoc test (TaqMan data, each time point compared to control (0 time point))

To determine how morphological tumor vessel changes manifest as molecular changes in gene expression, microarray analysis was performed on RNA from whole tumors treated with aflibercept for 8, 24 and 72 h. Mouse and human genes were assessed separately using mouse and human specific gene chips (custom Agilent microarray). Microarray analysis of mouse (host) genes in different tumors implied a rapid and consistent decrease in expression of a number of genes specific to endothelial cells [20–22] following aflibercept treatment. To confirm and extend the microarray findings, six genes were analyzed for expression changes in Colo205, C6 and HT1080 tumors treated with aflibercept for 8, 24 and 72 h by TaqMan, using primer pairs specific for murine mRNA. Gene expression was normalized to cyclophilin expression (similar results were obtained using GAPDH as a normalization gene; data not shown). Close inspection of these gene expression changes revealed two distinct temporal patterns: ‘acute’ and ‘delayed’ response genes. The ‘acute’ set of genes decreased in expression rapidly after aflibercept treatment (8 h) and remained decreased (Fig. 2a–c, black lines). Further, these ‘acute’ genes showed a large absolute decrease in expression levels, dropping up to 85 %. Among the ‘acute’ genes were potassium voltage-gated channel Isk-related subfamily gene 3 (Kcne3), endothelial cell-specific molecule 1 (Esm1) and nidogen2 (Nid2). Because of their rapid decrease after VEGF blockade (Fig. 2a–c, black lines), these ‘acute’ genes are likely direct targets of VEGF signaling.

A second set of genes decreased in expression at 24 and 72 h after aflibercept treatment, but were not yet significantly affected at the 8 h time point, thus showing a ‘delayed’ response (Fig. 2a–c, grey lines). Examples of genes that displayed robust ‘delayed’ changes were roundabout homolog 4 (Robo4), cadherin 5 (Cdh5) and tyrosine kinase with immunoglobulin-like and EGF-like domains 1 (Tie1). Other genes in this category included platelet/endothelial cell adhesion molecule 1 (Pecam1 or CD31) and intercellular adhesion molecule 2 (Icam2) (data not shown), two commonly used IHC endothelial cell markers [23, 24]. Thus, these ‘delayed’ genes may reflect a decrease in overall tumor vascularity or endothelial cell number. Changes in ‘delayed’ gene expression and MVD (Fig. 1d–i) appeared to have similar trends in terms of both timing and magnitude of decrease in different tumor types. When the combined expression of the ‘delayed’ gene changes was overlayed with MVD changes, comparable patterns emerged for each tumor (Fig. 2d–f), suggesting that ‘delayed’ gene changes can be used as markers for changed MVD in tumors treated with VEGF inhibitors.

### Decreased tumor perfusion following treatment with aflibercept

**Fig. 3:**
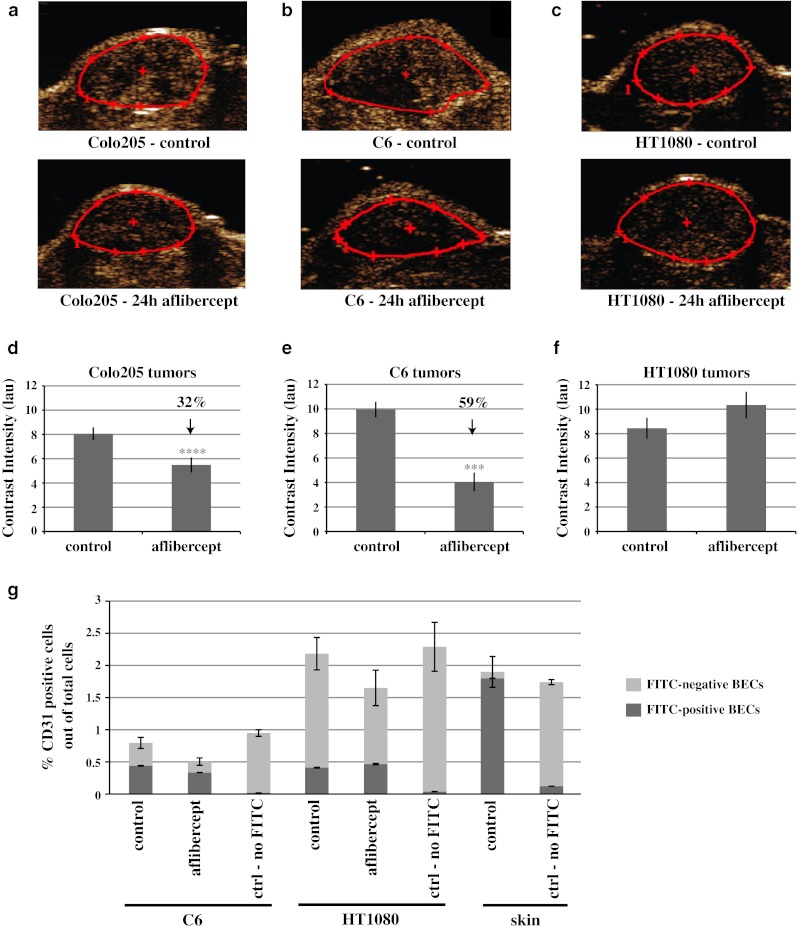
Perfusion decreased in response to aflibercept treatment in Colo205 and C6 tumors, but remained unchanged in HT1080 tumors. **a**–**c** Representative images of vessel perfusion assessed by 2-dimensional (2D) DCE-US in control and 24 h aflibercept-treated Colo205, C6 and HT1080 tumors. Tumors are outlined in *red*. **d**–**f** Quantitative analysis of vessel perfusion in control and 24 h aflibercept-treated Colo205 (n = 18 and 24, respectively), C6 (n = 8 and 12, respectively) and HT1080 tumors (n = 12 and 9, respectively) **g** Flow cytometry analysis of CD31-positive blood vessel endothelial cells (BECs) in combination with the intravenously injected perfusion marker FITC-lectin in control and 24 h aflibercept-treated C6 (n = 10 each treatment group; n = 5 for ‘no-FITC’ group) and HT1080 tumors (n = 10 each treatment group; n = 5 for ‘no-FITC’ group) as well as control-treated skin tissue (n = 5 each group). Shown are perfusion and flow cytometry results combined from multiple experiments. Results shown represent mean ± standard error of the mean (SEM). *P* < 0.05*, <0.01**, <0.001***, <0.0001**** by Mann–Whitney test (tumor perfusion). Differences between FITC-lectin positive BECs in ctrl versus aflibercept treated C6 or HT1080 tumors and between ctrl treated C6 and ctrl treated HT1080 tumors were not statistically significant by Mann–Whitney test

To determine whether VEGF blockade also affected vessel functionality, we assessed tumor vessel perfusion 24 h after aflibercept administration using contrast-enhanced micro-ultrasound. Analysis of 2-dimensional (2D) ultrasound data revealed that perfusion of Colo205 and C6 tumors decreased by 32 and 59 %, respectively (Fig. 3a, b, d, e). In comparison, HT1080 tumor perfusion was not decreased at 24 h after aflibercept treatment (Fig. 3c, f). Interestingly, although HT1080 tumors have a dramatically higher baseline MVD (55 %; Fig. 1i) than C6 (20 %; Fig. 1h) or Colo205 tumors (17 %; Fig. 1g), baseline perfusion in the three tumor types was comparable (relative contrast intensity values of 8–10; Fig. 3d–f, control), as was previously shown for other tumor types [25]. These data suggest that a smaller fraction of vessels are well perfused in HT1080 tumors compared to C6 or Colo205 tumors.

To further compare the relative amounts of perfused vessels in C6 and HT1080 tumors, vessel perfusion was assessed by another method, namely i.v. injection of FITC-conjugated *Lycopersicon esculentum* tomato lectin (FITC-lectin), which binds to the luminal surface of blood endothelial cells (BECs, defined as CD31 positive) in functionally perfused vessels. Following in vivo labeling, the proportion of endothelial cells in the tumor and normal skin, and the fraction of endothelial cells labeled by FITC-lectin were both assessed by flow cytometry. For reference, BECs from normal skin comprise 1.9 % of all skin cells, and 96 % of the BECs in normal skin were labeled by FITC-lectin (Fig. 3g, skin). As a further control, the same proportion of BECs were found in skin and tumors of mice that were injected with FITC-lectin versus those that were not injected, but virtually no BECs were found to be positive for FITC-lectin in non-injected mice (Fig. 3g).

The number of BECs in untreated C6 tumors (0.8 % of total cells) was significantly less than in HT1080 tumors (2.2 %) (Fig. 3g). Of the BECs in untreated C6 tumors, approximately 55 % were perfused (i.e., positive for FITC-lectin). In contrast, only 18 % of the BECs in untreated HT1080 tumors were perfused (Fig. 3g; Table 1). Thus, despite more than a twofold difference in total BEC, the fraction of BECs labeled by intravascular lectin (FITC-positive BECs) was similar in C6 and HT1080 tumors (0.40 vs. 0.43 % of total cells, respectively). This finding corroborates our micro-ultrasound findings that untreated C6 and HT1080 tumors have similar levels of perfusion as measured by micro-ultrasound (Fig. 3e, f, control), despite dramatically different MVD (Fig. 1i, h, control).

**Table 1:** Flow cytometry analysis of all CD31-positive blood vessel endothelial cells (BECs) and perfused (FITC-lectin positive) BECs derived from control and 24 h aflibercept-treated C6 and HT1080 tumors

	% CD31 + (BEC) cells out of total cells		Relative change (%)	% FITC-lectin + BECs out of total cells		Relative change (%)	% FITC-lectin + BECs out of total BECs	
	Control	Aflibercept		Control	Aflibercept		Control	Aflibercept
C6	0.8	0.5	−37	0.43	0.33	−24	55	66
HT1080	2.2	1.6	−28	0.4	0.48	+20	18	31

Treatment with aflibercept (24 h) decreased the number of BECs in C6 tumors to 0.5 % of total cells (~37 % decrease) and to 1.6 % in HT1080 tumors (~28 % decrease) (Fig. 3g, h; Table 1). These data correspond with the relative decrease in MVD after aflibercept treatment (Fig. 1h, i; Table 1). After 24 h of aflibercept treatment, the proportion of FITC-lectin positive BEC in C6 tumors increased slightly to 66 % of all BECs, although the total number of FITC-lectin positive BECs went down (to 0.33 % of total cells). In HT1080 tumors after aflibercept treatment, the proportion of FITC-lectin positive BEC also increased slightly to 31 % of all BECs, whereas the total number of BECs increased slightly (to 0.48 % of all cells) (Fig. 3g; Table 1). Again, these findings are consistent with perfusion changes seen by micro-ultrasound following treatment of these tumors with aflibercept (Fig. 3e, f). Thus, this flow cytometry-based analysis of tumor vessel perfusion provides a powerful link between functional perfusion assays and immunohistochemistry of tumor blood vessels following anti-VEGF treatment.

### Increased tumor hypoxia following treatment with aflibercept

**Fig. 4:**
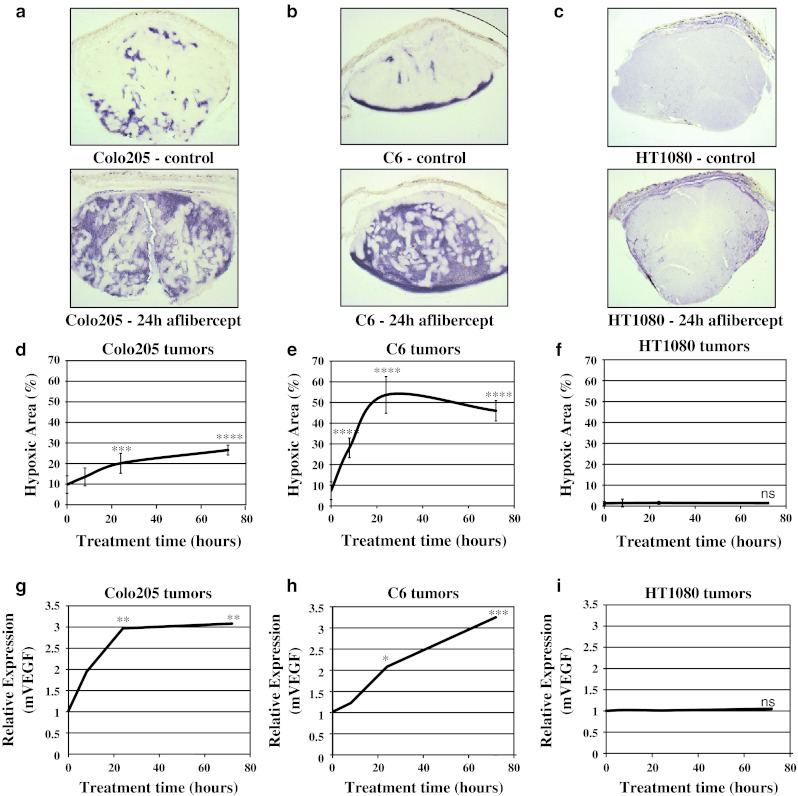
Tissue oxygenation decreased in response to aflibercept treatment in Colo205 and C6, but not in HT1080 tumors. **a**–**c** Representative images of hypoxia assessed by HypoxyProbe IHC in control and 24 h aflibercept-treated Colo205, C6 and HT1080 tumors (80 μm gelatin sections). **d-f)** Quantitative analysis of hypoxia area (%) in control and 8, 24 and 72 h aflibercept-treated Colo205, C6 and HT1080 tumors (n = 4–5 each time point/tumor type). **g**–**i** TaqMan analysis of the hypoxia responsive gene VEGF in Colo205, C6 and HT1080 tumors after 8, 24 and 72 h aflibercept treatment (n = 3–4 each time point/tumor type). All experiments were repeated at least twice; shown is an example experiment (n = 4–5 (hypoxia data) or n = 3–4 (TaqMan data) for each time point). Results shown represent means or mean ± standard deviation (SD). *P* < 0.05*, <0.01**, <0.001***, <0.0001**** by 1 way-ANOVA with Bonferroni post hoc test (hypoxia IHC, each time point compared to control (0 time point); TaqMan data, each time point compared to control (0 time point))

To determine whether the decreased tumor perfusion following aflibercept treatment resulted in tumor oxygenation changes, we analyzed hypoxia in Colo205, C6, and HT1080 tumors at 8, 24 and 72 h after aflibercept treatment. Hypoxia was assessed by HypoxyProbe IHC (Fig. 4a–c) as well as by analyzing the expression of VEGF, a hypoxia regulated gene (Fig. 4g–i). Colo205 and C6 tumors have hypoxic regions even under baseline conditions, which become more pronounced upon aflibercept treatment starting at 8 h (Fig. 4a, b, d, e). The increase in HypoxyProbe staining observed in C6 and Colo205 tumors after 24 h aflibercept treatment (Fig. 4d, e) corresponded with decreased perfusion (Fig. 3d, e). In comparison, HT1080 tumors had little or no hypoxic regions at baseline, and no increase in hypoxia at 72 h of treatment with aflibercept (Fig. 4c, f), consistent with the unchanged tumor perfusion (Fig. 3c, f). Similarly, expression of VEGF progressively increased in C6 and Colo205 tumors, whereas VEGF expression was unchanged in HT1080 tumors (Fig. 4g). Taken together, increased tumor hypoxia correlated with decreased tumor perfusion.

### Tumor perfusion changes correlated with long-term response to aflibercept

**Fig. 5:**
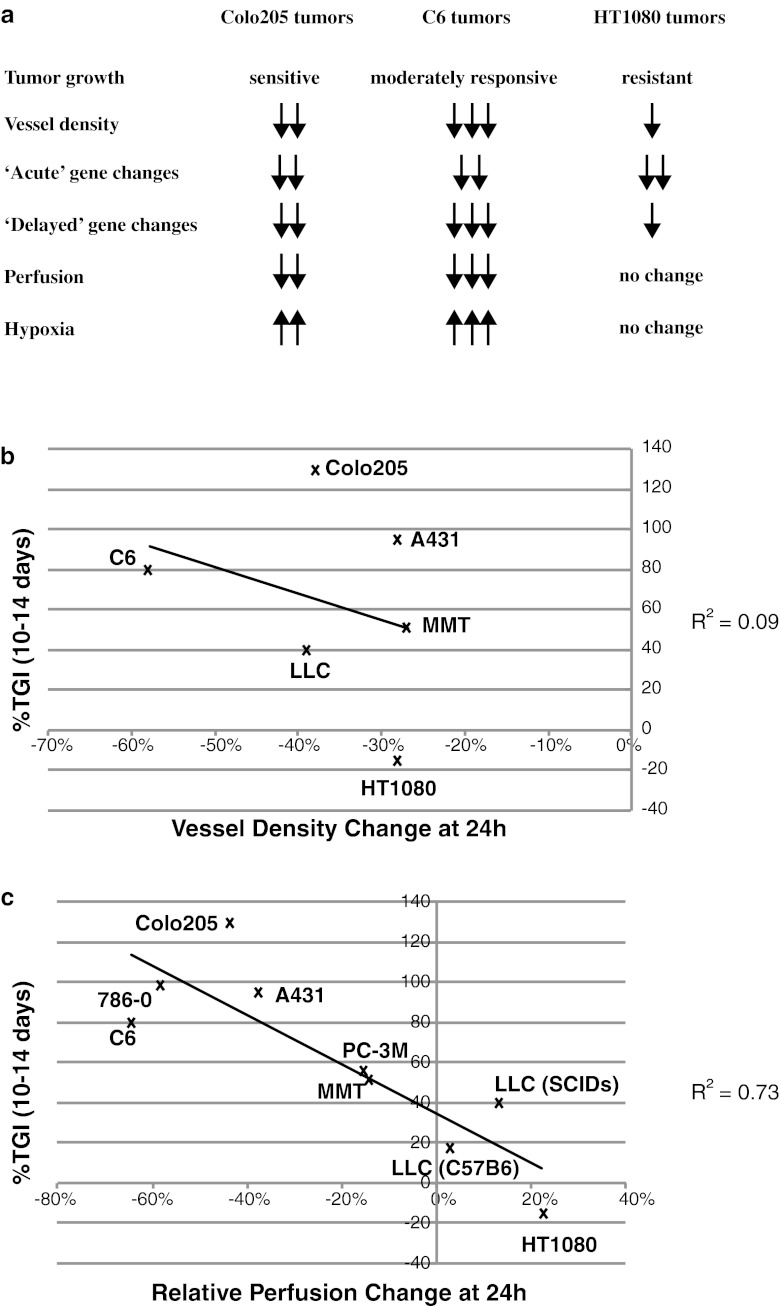
Changes in tumor perfusion, but not in MVD, 24 h after aflibercept treatment are predictive of long-term tumor growth inhibition. **a** Summary of aflibercept effects on long-term tumor growth and short-term (up to 72 h) MVD, gene expression, tumor perfusion and hypoxia. **b** Poor correlation between MVD changes (30 μm OCT sections; n = 4–5 each treatment group/tumor type) and long-term TGI (n = 5–7 each treatment group/tumor type) in Colo205, C6, HT1080, MMT, A431 and LLC tumors. **c** Good correlation between tumor perfusion changes (n = 7–24 each treatment group/tumor type) and long-term TGI (n = 5–7 each treatment group/tumor type) in Colo205, C6, HT1080, MMT, A431, LLC, 786-0 and PC-3 M tumors. All experiments were repeated at least twice; shown is an example experiment for tumor growth (n = 5–7) and vessel density (n = 4–5) data along with combined data for tumor perfusion data (n = 7–24 each treatment group/tumor type)

The results from our analysis of three tumor types suggested that rapid changes in tumor perfusion and/or hypoxia correlated better with long-term tumor growth response to aflibercept than did other parameters such as changes in microvessel density or vascular gene expression (Fig. 5a). To further assess whether tumor vascular perfusion changes at 24 h after aflibercept treatment correlated with long-term growth inhibition, we extended our analyses to several additional tumor types (A431, 786-0, MMT, LLC and PC-3 M) grown in immunocompromised SCID mice. We also included a syngeneic model, LLC tumors grown in C57Bl6 mice, to assess the effects of aflibercept on tumor perfusion and growth in immunocompetent mice. As expected, tumor growth inhibition in immunocompromised mice did not correlate well with changes in tumor vessel density (Fig. 5b, R^2^ = 0.09). In comparison, in this larger sample including one syngeneic model, tumor growth inhibition showed a correlation with changes in tumor perfusion (Fig. 5c, R^2^ = 0.73).

## Discussion

The search for early response and predictive biomarkers of tumor response to anti-angiogenic agents has so far not provided definitive candidates. While clinical studies have sought such markers by sampling numerous growth factors and cytokines, preclinical studies may be able to provide more mechanism-based candidates and approaches. In this study, we analyzed several tumors with a wide range of long-term tumor growth responses to anti-VEGF therapy. Using subcutaneous tumor models, we correlated early morphologic and functional vascular changes following treatment with aflibercept to long-term tumor growth inhibition (TGI). We found that changes in tumor hypoxia and perfusion correlated with long-term TGI, whereas changes in vascular gene expression and MVD showed a poor correlation.

In early clinical analyses, MVD was proposed as a prognostic indicator for disease stage, likelihood of metastasis, recurrence, and survival in a range of tumor types [26–28]. To date, however, neither baseline values, nor treatment-related changes in MVD have proven useful for evaluating or guiding anti-angiogenic treatments [29]. To extend the analysis of MVD, we identified a set of endothelial cell marker genes, including Tie1, Pecam1, Cdh5, Icam2 and Robo4 [20–22], which decreased following treatment with aflibercept. The timing and magnitude of the decrease in these genes correlated well with changes in tumor MVD. However, these gene expression changes did not correlate with long-term TGI. Further, changes in ‘acute’ gene expression, such as Esm1 and Nid2, which appear to reflect direct VEGF target genes [30–32], similarly did not correlate with long-term TGI following treatment with aflibercept. This latter finding suggests that VEGF inhibition within a tumor is a necessary but not sufficient determinant of efficacy of anti-VEGF therapy.

Agents that target other angiogenic signaling pathways further confound the attempts to correlate MVD, vascular markers or indicators of VEGF signaling with anti-tumor effects. For example, in pre-clinical models, blockade of the angiogenic ligand Dll4 results in increased MVD [18, 33] and endothelial cell marker genes (data not shown), but inhibits tumor growth, thus clearly showing that MVD changes are not predictive of anti-angiogenic treatment efficacy. In the case of Dll4 inhibition, the newly formed tumor vascular structures are non-functional [18, 33]. These findings, as well as our current results, emphasize the concept that changes in tumor vessel functionality are much more important for predicting tumor growth response than changes in the number of vessels, their morphology or their signaling profiles.

In preclinical models, intravenous injection of dyes like FITC-lectin, Hoechst 33342 or DiOC7 prior to sacrifice has frequently been used to distinguish perfused/functional vessels from non-perfused vessels in tissue sections [7, 19, 34–36]. In addition, FITC-lectin or Hoechst 33342 have been used with flow cytometry to detect perfused endothelial cells or to assess the ratio of tumor cells close to perfused blood vessels versus those further away [37, 38]. Our studies further validate the use of i.v. FITC-lectin combined with CD31 flow cytometry, to distinguish endothelial cells from perfused versus non-perfused vessels.

In clinical studies of VEGF blockade, DCE-MRI has been used frequently to evaluate the functional microvasculature within tumors. In particular, decreases in K^trans^, a volume transfer constant for contrast agent in blood/plasma and the extravascular extracellular space, was shown to be predictive of time to progression in liver cancer upon VEGF blockade [39]. Similarly, changes in K^trans^ allowed the prediction of responses in glioblastoma patients treated with bevacizumab and irinotecan [40]. In some preclinical tumor models, DCE-MRI has also revealed a decrease in K^trans^ in response to VEGF blockade [41]. However, DCE-MRI was not predictive of treatment efficacy upon anti-angiogenic therapy in other cancers, such as NSCLC [42]. In an attempt to better predict anti-angiogenic efficacy, DCE-MRI was combined with assessment of MVD and plasma collagen IV levels shortly (24 h) after the start of treatment. This ‘vascular normalization index’, was predictive of responsiveness to anti-angiogenic therapy in glioma patients [43]. In a follow-up study, a prolonged increase in tumor perfusion, as evidenced by DCE-MRI, was associated with longer survival in glioma patients [44]. However, a recent positron emission tomography (PET) imaging study reported a decrease in perfusion and impaired docetaxel delivery after a single dose of bevacizumab in NSCLC patients [14], suggesting that vessel normalization after VEGF blockade does not occur in NSCLC. In another recent study, single-photon emission computed tomography (SPECT) imaging revealed that anti-VEGF treatment decreased tumor uptake of an anti-Her2 antibody (trastuzumab) in preclinical breast cancer models, thus further supporting that VEGF blockade results in decreased tumor perfusion rather than vessel normalization [15].

In addition to DCE-MRI, PET and SPECT, other imaging modalities have been used to predict efficacy of anti-angiogenic therapies. For example, dynamic contrast-enhanced ultrasound (DCE-US) imaging has been used to assess tumor perfusion before and after treatment of various cancers with anti-angiogenic agents [45–47]. DCE-US imaging typically uses gas-filled lipid-shell microbubbles several micrometers in diameter as contrast agent [48]. DCE-US differs from DCE-MRI in that it solely assesses changes in vascular perfusion, while DCE-MRI measures a combination of blood flow through the vasculature as well as tracer movement across the vessel wall [49]. Although DCE-MRI and DCE-US appear to have predictive potential in anti-angiogenic therapy, DCE-US may be less sensitive to changes in tumor vascular permeability, and thus be more robust for assessing changes in tumor perfusion.

Decreases in tumor perfusion can result in hypoxia, as was observed after aflibercept treatment of sensitive Colo205 and moderately responsive C6 tumors, but not in resistant HT1080 tumors. These findings can be compared to the vascular normalization hypothesis, which proposed that tumor vessels remaining after anti-VEGF therapy temporarily ‘normalize’ in terms of morphology and functionality, resulting in increased tumor blood flow and decreased hypoxia [10]. Other preclinical studies, however, have shown that the anti-angiogenic agents DC101 and AG-013736 induce decreased perfusion and increased hypoxia [13, 35, 50]. Although it is well established that human tumors are often hypoxic and poorly perfused [51], direct measurement of tumor oxygenation before and after VEGF blockade in patients is challenging. Instead, hypoxia changes have been assessed indirectly. For example, bevacizumab treatment of RCC patients resulted in increased tumor cell apoptosis, along with increased tumor cell proliferation, which were hypothesized to be at least partially due to increased blood flow and decreased hypoxia [52].

The current study used various tumor models grown subcutaneously in mice, a site that can be readily accessed for micro-ultrasound studies of tumor perfusion. While the vascular structures and responses to anti-angiogenic therapies of such tumors may not fully reflect those of primary and metastatic human tumors, the ability to directly measure tumor blood flow provides opportunities to identify potential early response biomarkers that can be further tested in orthotopic preclinical tumor models and in clinical settings.

Early response biomarkers that can predict long-term outcome to therapy would be powerful tools, and panels of such potential biomarkers for anti-angiogenic therapies have been explored. For example, changes in circulating VEGF or PlGF levels, as well as tumor VEGF levels, were thought to be predictive, but to date have not shown to be well correlated with outcome [53]. In the current preclinical study, decreases in tumor perfusion and increases in hypoxia following treatment of subcutaneous xenograft and syngeneic models with aflibercept correlated with long-term TGI. Our results suggest that perfusion changes, as measured by DCE-US, shortly after treatment with VEGF inhibitors or possibly other anti-angiogenic therapies, could potentially be used as an early response biomarker to assess treatment efficacy.
